# Increasing cell density globally enhances the biogenesis of Piwi-interacting RNAs in *Bombyx mori* germ cells

**DOI:** 10.1038/s41598-017-04429-7

**Published:** 2017-06-23

**Authors:** Shozo Honda, Phillipe Loher, Keisuke Morichika, Megumi Shigematsu, Takuya Kawamura, Yoriko Kirino, Isidore Rigoutsos, Yohei Kirino

**Affiliations:** 1 Computational Medicine Center, Sidney Kimmel Medical College, Thomas Jefferson University, Philadelphia, Pennsylvania USA; 2 Department of Biomedical Sciences, Cedars-Sinai Medical Center, Los Angeles, California, USA

## Abstract

Piwi proteins and their bound Piwi-interacting RNAs (piRNAs) are predominantly expressed in the germline and play crucial roles in germline development by silencing transposons and other targets. *Bombyx mori* BmN4 cells are culturable germ cells that equip the piRNA pathway. Because of the scarcity of piRNA-expressing culturable cells, BmN4 cells are being utilized for the analyses of piRNA biogenesis. We here report that the piRNA biogenesis in BmN4 cells is regulated by cell density. As cell density increased, the abundance of Piwi proteins and piRNA biogenesis factors was commonly upregulated, resulting in an increased number of perinuclear nuage-like granules where Piwi proteins localize. Along with these phenomena, the abundance of mature piRNAs also globally increased, whereas levels of long piRNA precursor and transposons decreased, suggesting that increasing cell density promotes piRNA biogenesis pathway and that the resultant accumulation of mature piRNAs is functionally significant for transposon silencing. Our study reveals a previously uncharacterized link between cell density and piRNA biogenesis, designates cell density as a critical variable in piRNA studies using BmN4 cell system, and suggests the alteration of cell density as a useful tool to monitor piRNA biogenesis and function.

## Introduction

Piwi proteins and their bound Piwi-interacting RNAs (piRNAs) are abundantly expressed in animal germlines to regulate transposable elements and other targets such as germline mRNAs^1–3^. The indispensability of the Piwi/piRNA function in germline development has been demonstrated by studies on animals lacking Piwi or other piRNA biogenesis factors that show elevated transposon levels and defects in gametogenesis, eventually resulting in sterility^1–3^. piRNAs are 24–31 nucleotides (nt) in length and show a highly complex mix of sequences with immense diversity. The current model for piRNA biogenesis proposes two distinct piRNA biogenesis pathways: primary and secondary. In the primary pathway, single-stranded RNAs are transcribed from defined genomic regions called piRNA clusters^4, 5^ and are likely fragmented into shorter precursor piRNAs by involvement of Zucchini endonuclease and other unknown proteins^6–8^. The precursor piRNAs are then loaded onto Piwi proteins for 3′-end formation by a Trimmer exonuclease^9–11^ or by Zucchini^12–16^, followed by 3′-end methylation by Hen1 methyltransferase^17–21^. The generated primary piRNAs are then subjected to the secondary biogenesis pathway, known as the ping-pong amplification loop, in which primary piRNAs complexed with Piwi proteins cleave complementary target RNAs for abundant secondary piRNA production^4, 22^.

A stumbling block in studying the piRNA biogenesis has been the lack of suitable cell culture systems, because Piwi proteins and piRNAs are expressed predominantly in germlines which are not readily available for cell culturing. However, some culturable cell lines, such as *Bombyx mori* BmN4 cells^23^, *Drosophila* female germ-line stem cells/ovarian somatic sheet (fGS/OSS) and their deriving ovarian somatic cells or sheet (OSC/OSS)^24–27^, and *Drosophila* Kc167 cells^28^, have been noted to endogenously equip the piRNA pathway, thereby providing researchers with a convenient system for gene expression or silencing by transfection of DNA or siRNA.

BmN4 cells are the only reported germ cells that express an endogenous piRNA pathway. In the cells, both of the two *Bombyx* Piwi proteins, Siwi and BmAgo3, and their bound piRNAs are expressed^23^, which function to silence transposons and cleave piRNA complementary targets^29–32^. BmN4 cells possess fully functional primary and secondary piRNA biogenesis pathways and have contributed to elucidating the molecular mechanisms underlying them^9, 10, 13, 30, 32–40^. Various protein factors have been shown to be involved in the piRNA biogenesis pathways in BmN4 cells. Hsp90 is suggested to facilitate Piwi turnover by eliminating ping-pong cycle byproducts^33^ and is also required for piRNA precursor loading to Piwi proteins^34^. PNLDC1 Trimmer catalyzes the 3′-terminal formation of piRNAs^9, 10^ and BmPapi supports the reaction^36^. BmVasa functions as a core of the “piRNA Amplifier” protein-RNA complex which plays a significant role in the ping-pong amplification cycle^37^. BmSpn-E and BmQin form a dimer that is required for primary piRNA biogenesis^38^.

In various cell culture systems, cell–cell contact regulates cell migration, proliferation, and differentiation^41–43^. Cell–cell contact is also reported to regulate the biogenesis pathway of microRNA (miRNA)^44^. Here we examined whether cell density regulates the piRNA pathway using BmN4 cells. Specifically, we investigated the expression levels of Piwi proteins and other piRNA biogenesis protein factors, piRNA precursor, mature piRNAs, and transposons in the cells with different densities. The obtained results collectively suggest that increasing cell density promotes piRNA biogenesis and that the resultant accumulation of mature piRNAs is functionally significant for transposon silencing. These results reveal a previously uncharacterized link between cell density and piRNA biogenesis, uncovering a critical parameter that should be taken into consideration in piRNA studies. Moreover, our results suggest that the alteration of BmN4 cell density should provide a useful tool to monitor piRNA biogenesis and function.

## Results

### The abundance of Piwi proteins and piRNA biogenesis factors is linked to cell density

**Figure 1:**
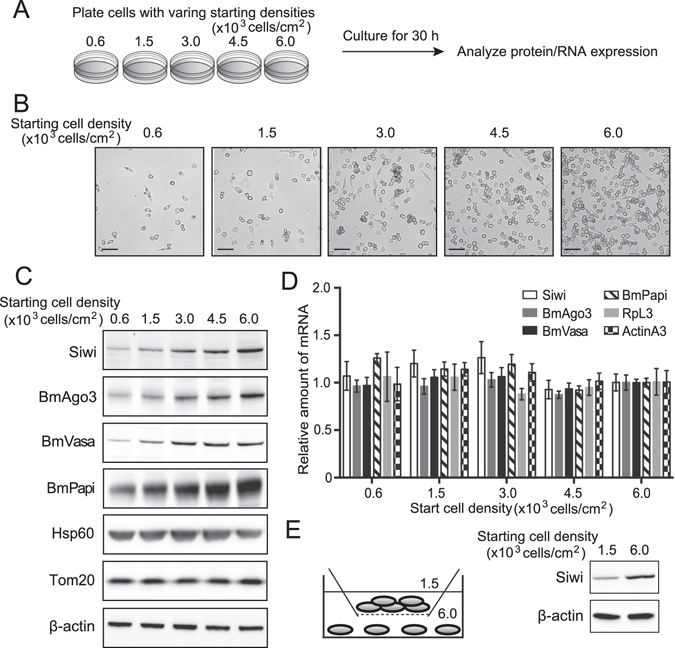
Cell density-dependent change in the levels of Piwi proteins and piRNA biogenesis factors. (**A**) Schematic representation of the experimental design. BmN4 cells were plated at the represented cell densities, cultured for 30 h, and then harvested for expression analyses of protein and RNA. (**B**) The representative DIA images (magnification ×40) of the cells with the indicated starting cell densities. Scale bar, 100 μm. (**C**) Total protein lysates were probed on Western blots with indicated antibodies. (**D**) mRNA transcripts encoding the indicated proteins [Ribosomal protein L3 (RpL3) and ActinA3: control] were quantified by qRT-PCR. Ribosomal protein 49 (Rp49) was used as an internal control^36^. mRNA levels in the cells with 6.0 × 10^3^ cells/cm^2^ starting density were set as 1. Each data set represents the average of three independent experiments with bars showing the SD. (**E**) The low- and high-density cells, which were co-cultured in the same medium using a transwell system, were subjected to Western blots with indicated antibodies.

During the course of investigating the piRNA pathway using BmN4 cells, we noticed that the levels of Piwi proteins and piRNAs differed in cells with varying densities. To clarify the relationship between cell density and the piRNA pathway, we plated BmN4 cells with varying cell densities, cultured them for 30 h, and then harvested the cells for the analyses of protein and RNA expression (Fig. 1A and B). Western blots, with quantitative ability (Supplementary Fig. S1 and S2), showed that the abundance of both of the Piwi proteins, Siwi and BmAgo3, as well as the factors involved in piRNA biogenesis, BmVasa and BmPapi, commonly increased along with increasing cell density (Fig. 1C). No obvious quantitative changes were observed in the control cytoplasmic (β-actin) and mitochondrial (Hsp60 and Tom20) proteins (Fig. 1C), in total protein staining patterns (Supplementary Fig. S3), and in mitochondrial staining patterns (Supplementary Fig. S4), suggesting that the increases in expression levels may be specific to the protein factors involved in piRNA biogenesis.

To examine whether the increased accumulation of piRNA biogenesis-related factors is caused by transcriptional upregulation of their mRNAs, we quantified their mRNA expression levels by qRT-PCR. As shown in Fig. 1D, none of their mRNA levels was affected by cell density, implying that the increased accumulation of the piRNA biogenesis proteins with increasing cell density occurs through post-transcriptional mechanisms, such as translational activation or increased protein stability.

The observed increase in accumulation of piRNA biogenesis-related factors could be triggered by one of many potential events occurring in the cells with increased cell density. The different expression levels of Siwi in high- and low-density cells were retained when the cells with the two densities were cultured in the same medium using the Transwell system (Fig. 1E), indicating that the increased accumulation in high-density cells is not due to the confounding effects of nutrients or other diffusible factors in the cultured medium. In addition, although the increase of cell density accelerates the rate of cell proliferation (Supplementary Fig. S5A), the Piwi protein levels are not affected in the cells in which the cell cycle was arrested by double thymidine block (Supplementary Fig. S5B and C), implying that the increased cell proliferation rate is not a significant factor for the increased accumulation of piRNA biogenesis factors in high-density cells. These results suggest that physical cell–cell contact, but not cultured medium or cell proliferation, contributes to the upregulation of the abundance of piRNA biogenesis factors.

### Cell density-dependent formation of perinuclear nuage-like granules

**Figure 2:**
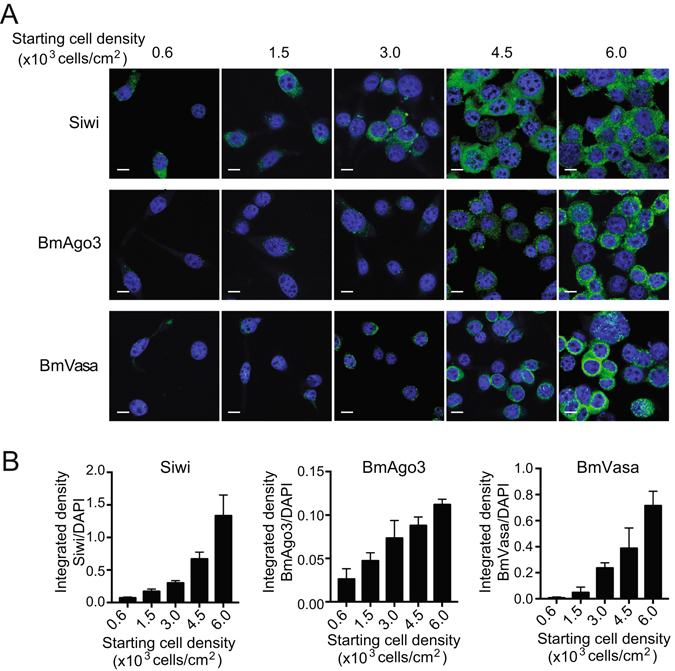
Cell density-dependent formation of perinuclear granules. (**A**) Immunofluorescence staining of the indicated proteins in BmN4 cells with different densities is shown in green. DNA was counterstained with DAPI in blue. Scale bar, 10 μm. (**B**) The intensities of fluorescence signals from each protein were quantified and normalized to those from DAPI. Each data set represents the average of three image panels with bars showing the SD.

Piwi proteins accumulate in amorphous, ribonucleoprotein-rich, perinuclear granules that are named nuage in *Drosophila* and intermitochondrial cement or chromatoid body in mice^4, 45^. In BmN4 cells, Siwi, BmAgo3, and BmVasa are mainly co-localized in perinuclear nuage-like granules^33, 36–38^ (Supplementary Fig. S1B and C). To examine the influence of cell–cell contact on the granule formations, the three proteins were immuno-stained in cells of varying densities. As shown in Fig. 2A and B, the number of perinuclear granules and protein signal intensities drastically increased along with increasing cell density, suggesting that increasing cell density promotes granule formation and accumulation of the piRNA biogenesis factors in the granules.

### Global increase in mature piRNA abundance with increasing cell density

**Figure 3:**
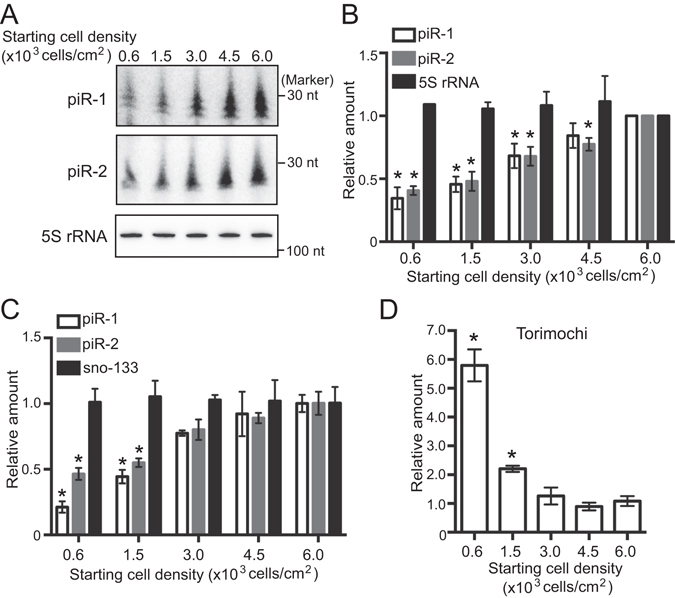
Cell density-dependent change in the levels of piRNAs and their precursors. (**A**) The expression levels of piR-1, piR-2, and 5 S rRNA in BmN4 cells with different densities were analyzed by Northern blot. (**B**) The Northern blot bands were quantified and shown as relative abundances to signal intensities from the cells with 6.0 × 10^3^ cells/cm^2^ starting density (set as 1). Each data set represents the average of three independent experiments with bars showing the SD. The asterisks indicate significant difference (P < 0.01, Welch’s t-test) between the cells with 6.0 × 10^3^ cells/cm^2^ starting density and those with other starting densities. (**C**,**D**) piRNAs and sno-133 (**C**) and Torimochi piRNA precursor (**D**) were quantified by qRT-PCR. 5 S rRNA was used as an internal control. Expression levels in the cells with 6.0 × 10^3^ cells/cm^2^ starting density were set as 1. Each data set represents the average of four independent experiments with bars showing the SD. The asterisks indicate significant difference (P < 0.01, Welch’s t-test) between the cells with 6.0 × 10^3^ cells/cm^2^ starting density and those with other starting densities.

We subsequently investigated the expression levels of mature piRNAs in BmN4 cells with varying densities. Northern blot for the piR-1 and piR-2, which specifically bind to Siwi and BmAgo3, respectively^36^, showed that the expression levels of both piRNAs were commonly enhanced by increasing cell density (Fig. 3A and B). The results were confirmed by piRNA quantification using qRT-PCR with a stem-loop primer (Supplementary Fig. S6), which was modified from stem-loop qRT-PCR for miRNA quantification^46^. As observed in Northern blot, qRT-PCR quantification revealed increased accumulation of both piR-1 and piR-2 in high-density cells (Fig. 3C), suggesting that the abundances of both the Siwi- and BmAgo3-bound piRNAs, as well as the Siwi and BmAgo3 proteins, are linked to cell density. Because increasing cell density has been shown to globally up-regulate the expression of miRNAs in human cancer cells^44^, we also quantified the levels of miRNAs. All 6 examined miRNAs commonly showed enhanced accumulation in high-density cells (Supplementary Fig. S7), suggesting that not only piRNAs but also miRNAs are under control of cell density.

To investigate whether piRNA precursors are also regulated by cell density, we quantified a reliable piRNA precursor expressed from *Torimochi*, a representative functional piRNA cluster in the *Bombyx* genome^35^. The Torimochi piRNA precursor contains a 5′-Cap and 3′-Poly(A)-tail, and produces abundant piRNAs in BmN4 cells^35^. In contrast to those of piRNAs, the expression levels of the Torimochi precursor decreased as cell density increased (Fig. 3D). We reason that the decreased levels could result from global enhancement of piRNA biogenesis mechanisms in higher density cells and increased use of precursors for piRNA production.

**Figure 4:**
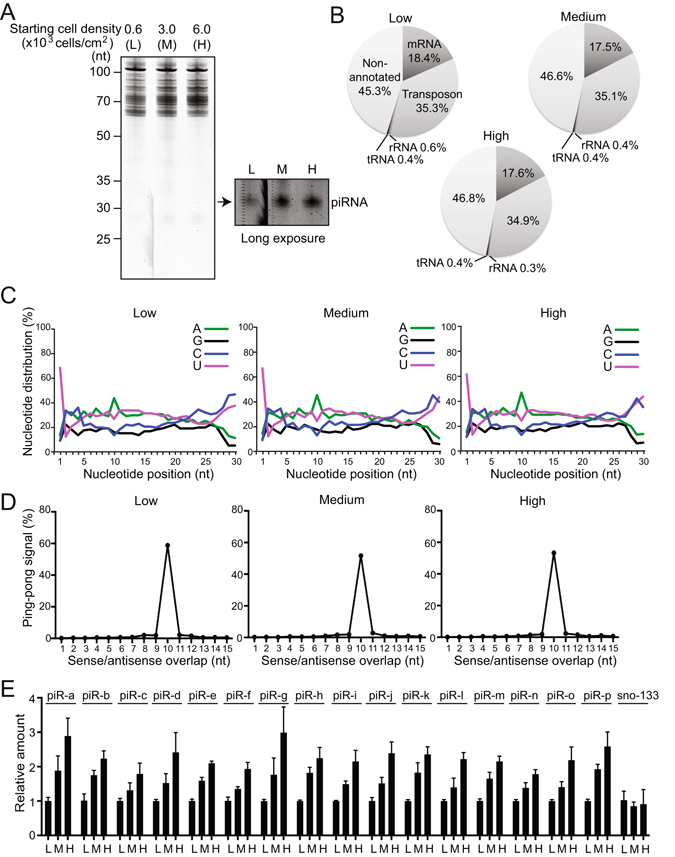
Sequence analyses of piRNAs expressed in the cells with different densities. (**A**) Total RNA (7 μg) extracted from BmN4 cells with the 0.6 (low), 3.0 (medium), or 6.0 × 10^3^ (high) cells/cm^2^ starting densities was separated by denaturing urea-PAGE and stained using SYBR-Gold. (**B**) Pie charts summarizing the annotations of the *B*. *mori* genome-mapped piRNA-enriched reads (24–30 nt) obtained from the SOLiD NGS for the small RNAs of the low-, medium-, or high-density cells. Because non-unique mapping was performed, the existence of double-counted reads from annotation overlaps made the number of total annotated and ﻿non-annotated reads higher than that of total mapped reads. The total annotated/non-annotated reads were used as the denominator. (**C**) Nucleotide composition of the piRNA-enriched reads from the low-, medium-, or high-density cells charted with the X-axis representing the nucleotide position relative to the 5′-ends of the reads. (**D**) The distances between 5′-ends of the piRNAs across opposite genomic strands were plotted (ping-pong analysis). Approximately 55% of reads obtained from the low-, medium-, or high-density cells commonly conformed to a separation preference of 10 nt (ping-pong signal). (**E**) The expression levels of 16 different piRNAs (piR-a–p) abundantly present in the sequencing data and sno-133 (control) were quantified by qRT-PCR. 5 S rRNA was used as an internal control. The expression levels in the low-density cells were set as 1. Each data set represents the average of three independent experiments with bars showing the SD.

To globally assess the influence of cell density on piRNA populations, we performed SOLiD next-generation sequencing (NGS) of small RNA species of BmN4 cells with low, medium, or high densities. In staining patterns of total RNA extracted from the respective cells, we confirmed the increasing abundance of ~27–29-nt mature piRNA populations in higher density samples, whereas other RNAs (e.g., tRNAs) showed no apparent quantitative differences between samples (Fig. 4A). NGS yielded approximately 118, 145, and 152 million total reads from the low, medium, and high samples, respectively. After mapping to the *B*. *mori* genome, majority of the 16–50-nt reads (87.5%, 90.1%, and 92.8% of the reads from the low, medium, and high samples, respectively) were derived from the regions of miRNAs (19–23 nt) and piRNAs (24–30 nt). Given that not only piRNAs but also miRNAs showed increased levels in high-density cells, quantitative comparison of the reads between the samples was not possible due to a lack of a normalization method. Consequently, we utilized the NGS data for qualitative comparison of the reads and for comparison of piRNA populations. The relative proportions of known annotations of the reads were not appreciably varied between samples (Fig. 4B). Nucleotide composition analyses showed strong piRNA characteristic biases for uridine on the 5′-end (position 1) and adenine on the position 10, but no difference was observed between samples (Fig. 4C). Moreover, in the low, medium, and high samples, we detected similar patterns of ping-pong signals (sense-antisense piRNA pairs overlapping by 10 nt at their 5′-ends that are characteristic of a ping-pong amplification cycle in secondary piRNA biogenesis^4^) (Fig. 4D). Mapping patterns of the reads to transposon sequences were also similar between samples (Supplementary Fig. S8). To confirm the global influence of cell density on piRNA abundance, we selected 16 mature piRNAs (Supplementary Table S2) from the reads meeting the following criteria: (1) 27–29 nt in length, (2) among the top 100 abundant reads in at least two of the three libraries (low, medium, and high), and (3) among Siwi- or BmAgo3-bound piRNAs sequenced in our previous study^36^. qRT-PCR quantifications of the selected piRNAs in low, medium, and high samples revealed that all 16 examined piRNAs were invariably upregulated along with the increase of cell density (Fig. 4E). Considered collectively, these results suggest that increasing BmN4 cell density globally activates the piRNA biogenesis mechanism via both primary and secondary pathways, resulting in the widespread accumulation of piRNAs in higher-density cells.

### Cell density-dependent transposon silencing

**Figure 5:**
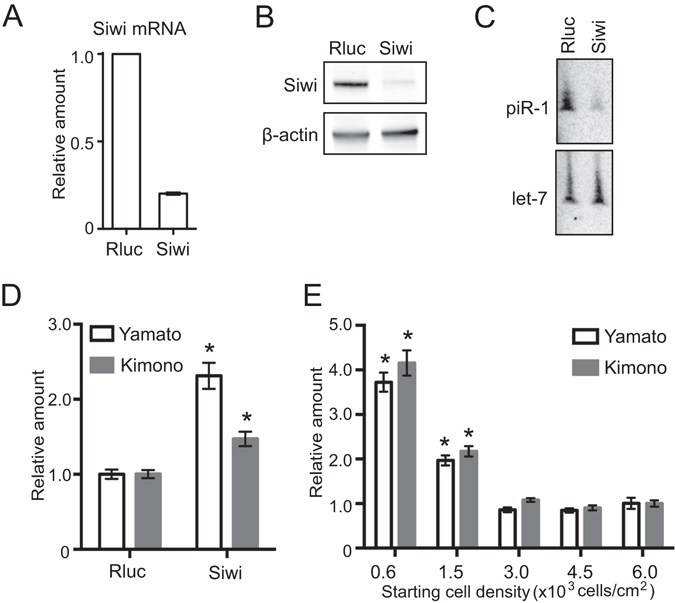
Cell density-dependent change in the transposon levels. (**A**) Siwi mRNA from BmN4 cells treated with dsRNA targeting Renilla luciferase (Rluc, negative control) or Siwi was analyzed by qRT-PCR. Rp49 was used as an internal control, and the level in the Rluc dsRNA-treated cells was set as 1. The average of three independent experiments with bars showing the SD is shown. (**B**) The levels of Siwi and β-actin (control) in the Rluc- or Siwi-depleted cells were analyzed by Western blot. (**C**) The levels of piR-1 and let-7 miRNA (control) in the Rluc- or Siwi-depleted cells were analyzed by Northern blots. (**D**,**E**) The expression levels of Yamato and Kimono transposons in the Rluc- or Siwi-depleted cells (**D**) and the cells with the indicated starting densities (**E**) were quantified by qRT- PCR. The expression levels in the Rluc-depleted cells or the cells with 6.0 × 10^3^ cells/cm^2^ starting density were set as 1. Each data set represents the average of three independent experiments with bars showing the SD. The asterisks indicate significant difference (P < 0.01, Welch’s t-test) from the standard cells.

As cell density regulates the abundance of piRNAs, which function to silence transposons, we investigated whether cell density affects the piRNA-mediated transposon silencing. To explore and identify the transposon species whose expressions are well regulated by piRNAs in BmN4 cells, we knocked-down Siwi expression and analyzed the eight transposons (*Yamato*, *Kimono*, *Ichiro*, *Yokohama*, *Noguchi*, *Yaocho*, *Kimutaku*, and *Nukegara*) in which antisense strands produce abundant Siwi-bound piRNAs^23^. As in our previous study, RNAi-mediated silencing of Siwi reduced the expression levels of Siwi mRNA (Fig. 5A), Siwi protein (Fig. 5B), and Siwi-bound piR-1 (Fig. 5C). Among the eight transposons, *Yamato* and *Kimono* showed clear derepression upon the reduction of Siwi/piRNA complexes (Fig. 5D), indicating that *Yamato* and *Kimono* are the transposons whose expressions are well silenced by the piRNA pathway in BmN4 cells. The expression levels of both Yamato and Kimono decreased as cell density increased (Fig. 5E), which corresponds to the increase of piRNA abundance in increased cell density. These results suggest that cell density-dependent piRNA biogenesis and its resultant piRNA accumulation indeed have functional significance for the silencing of transposon expressions.

## Discussion

**Figure 6:**
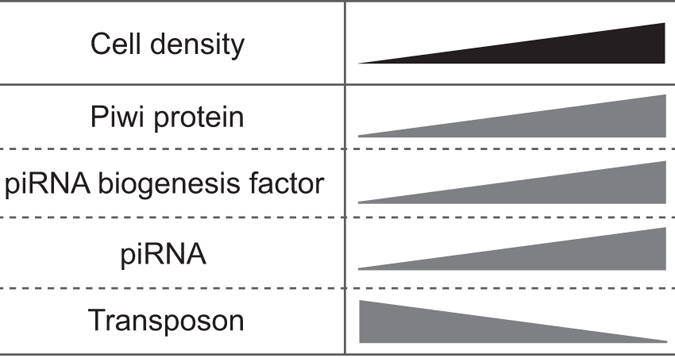
Cell density is linked to piRNA biogenesis and function. As BmN4 cells are cultured with increasing cell density, Piwi proteins, piRNA biogenesis factors, and mature piRNAs are increasingly accumulated, whereas levels of transposons are reduced.

Here we demonstrated that the piRNA pathway is intimately associated with cell density in *Bombyx* BmN4 cells. As the cells are cultured with increasing cell density, Piwi proteins, piRNA biogenesis factors, and mature piRNAs are increasingly accumulated, whereas levels of a long piRNA precursor and transposons are reduced (Fig. 6). The consistent mRNA levels of Piwi proteins and piRNA biogenesis factors despite varying cell densities (Fig. 1D) suggest that their protein levels are regulated through a cell-density-linked post-transcriptional event. One of the possible post-transcriptional events would be the enhancement of protein stability by forming complexes in the perinuclear nuage-like granules, as the formation of the granule is also dependent on cell density (Fig. 2). One of the examined piRNA biogenesis factor, BmPapi, is localized on the outer membrane of the mitochondria^36^, yet increasingly accumulates in high-density cells. Therefore, cell density-dependent accumulation is a common feature of all four of the examined piRNA biogenesis-related factors localizing in different locations in BmN4 cells (Fig. 1C). The increase of piRNA biogenesis factors could promote piRNA production from precursors, which could be the reason for the decreased levels of a piRNA precursor (Fig. 3C). As a result, mature piRNA levels are increased (Figs 3 and 4), eventually resulting in increased repression of transposon expression in the higher density cells (Fig. 5E). Although the levels of miRNAs, as well as piRNAs, are increased in high-density cells, cell density-dependent regulatory pathway for piRNAs and miRNAs might be distinct, because, in human cancer cells, cell density regulates miRNA levels through Drosha activity but does not affect the levels of Argonatue proteins^44^.

The cell density-dependent regulation of the piRNA pathway was not caused by the changes in nutrients or other diffusible factors in the culturing medium (Fig. 1E). Although cell density is known to affect cell proliferation^41, 47^, and indeed, the increased density of BmN4 cells enhanced cell proliferation (Fig. S5A), our cell cycle arrest experiments suggested that cell proliferation rate is not directly associated with the piRNA pathway (Fig. S5B and C). Therefore, we reason that physical cell–cell contact could be the factor contributing to the regulation of the piRNA pathway. Cell–cell contact-dependent signals fulfill key and fundamental roles in animal development and cell differentiation^48, 49^. For example, it is well known that E-cadherin-mediated cell–cell contact and subsequent maturation of adherens junctions with β-catenin mediate the Wnt signaling pathway^50, 51^. Our results are in particular reminiscent of the regulation of Piwi expression through cell–cell communications in the *Drosophila* stem cell niche where the supporting cells exist to strictly control the maintenance and differentiation of germline stem cells^49^. In the niche, the Bruton’s tyrosine kinase 29 A phosphorylates β-catenin under Wnt signaling pathway, which enhances Piwi protein expression to terminate germ cell proliferation^52^. Given these reports, our observations might not just represent an artificial phenomenon specific to BmN4 cells, but rather reflect the important regulatory mechanisms of the piRNA pathway in the context of germline development in organisms.

BmN4 cells are the only reported germ cells equipping the endogenous piRNA pathway, and they are therefore widely used as a cell line system to analyze piRNA biogenesis and function. Our findings indicate that cell density is a critical variable that should be closely monitored for accurate analyses of piRNA expression, biogenesis, and function in cell culture settings. Moreover, our findings suggest the utilization of cell culturing with different cell densities as a unique tool to monitor piRNA biogenesis. To date, the mechanism underlying the production of piRNA precursors and their processing into mature piRNAs have remained elusive. Although the use of various knockout or knockdown organisms with depleted expression of targeted piRNA biogenesis factors has greatly contributed to clarifying piRNA biogenesis and function, these studies always observed the “end point” resulting from the factor depletion, and they suffered limitations in monitoring the intermediate state of the phenomenon. As gradual regulation of the piRNA pathway is achieved by changing culturing cell density, RNA-seq and proteomic analyses of BmN4 cells with different cell densities might serve as a useful system for the analyses of piRNA precursors, piRNA biogenesis factors, and formation of perinuclear germ granules.

## Methods

### BmN4 cell culture

BmN4 cells were cultured at 27 °C in Insect-Xpress medium (Lonza). To analyze protein and RNA expressions in the cells of varying densities, BmN4 cells were plated at the densities of 0.6 × 10^3^, 1.5 × 10^3^, 3.0 × 10^3^, 4.5 × 10^3^, or 6.0 × 10^3^ cells/cm^2^. After 30 h incubation, the cells showing confluences of approximately 10, 25, 50, 75, or 100% were harvested. For assessment of the influence of diffusible factors in medium, the cells with 1.5 × 10^3^ and 6.0 × 10^3^ cells/cm^2^ starting densities were co-cultured for 30 h in the same medium using Polycarbonate Membrane Transwell® Inserts with 0.4 μm membrane pore size (Corning).

### Western blot

Western blot using BmN4 cell lysates was performed as described previously^36^. Cell lysates were prepared from identical number of the cells from respective density conditions in a lysis buffer containing 20 mM Tris-HCl pH 7.4, 200 mM NaCl, 2.5 mM MgCl_2_, 0.5% NP-40, 0.1% Triton X-100, and complete protease inhibitor (Roche Diagnostics). After quantifying protein concentration of the lysates, 20 μg of each lysate was loaded onto SDS-PAGE. The following antibodies were used: S213 anti-Siwi^36^, anti-BmAgo3^23^, anti-Tom20 (Santa Cruz Biotechnology), anti-Hsp60 (Cell Signaling), anti-β-actin (Abcam), anti-BmPapi^10^, and BmVasa571 anti-BmVasa^53^. The anti-BmVasa is able to specifically recognize BmVasa in Western blot and immunofluorescence (Supplementary Fig. S1). The Western blot band intensities showed clear linearity to the amount of lysate input (2.5–20 μg), suggesting the quantification ability of the Western blot analyses (Supplementary Fig. S2).

### Immunofluorescence and confocal microscope

BmN4 cells were plated on a slide glass chamber (Lab-Tek) with differing densities and incubated for 30 h. Immunofluorescence staining was performed as described previously^36^ using anti-Siwi (diluted 1:2000), anti-BmAgo3 (1:5000), and anti-BmVasa (1:1000) as primary antibodies. Alexa Fluor 488 goat anti-rabbit IgG (Life Technologies) was used as a secondary antibody. After DNA counterstaining with ProLong Gold Antifade Reagent with DAPI (Life Technologies), images shown in Fig. 2A and Supplementary Fig. S1B were acquired using a Leica SP5 confocal microscope and a Nikon Eclipse Ti-U microscope, respectively. In Fig. 2B, signal intensities of each protein were quantified using ImageJ software. The color images were divided to two images (target protein and DAPI) by running split channel, and, after running despeckle to remove noises, segmentations were performed by setting the following threshold values: Siwi, 100–255; BmAgo3, 50–255; BmVasa, 100–255; and DAPI: 30–255. The integrated intensities of target proteins were then measured and normalized to those of DAPI.

### qRT-PCR for mRNAs, piRNA precursor, and transposons

Total RNAs were extracted from identical number of the cells from respective density conditions using TRIsure (Bioline). The use of identical cell amounts as starting materials would avoid a potential bias of RNA extraction efficiency^54^ between samples. After treatment with RQ1 DNase (Promega), target sequences were reverse-transcribed in the DNase-treated total RNA using each gene-specific reverse primer (Supplementary Table S1) by using SuperScript III reverse transcriptase (Life Technologies). The resultant cDNA was quantified by real-time PCR with forward and reverse primers (Supplementary Table S1) using the CFX96 Real-Time Detection System (Biorad) and Ssofast Evagreen Supermix (Biorad).

### qRT-PCR for mature piRNAs using stem-loop reverse primers

Mature piRNAs were quantified by modifying a quantitative stem-loop RT-PCR method which has been widely used for miRNA quantification^46^. For piRNA-1 (piR-1) and piRNA-2 (piR-2), to reverse-transcribe mature piRNA, 100 ng of the DNase-treated BmN4 total RNA was incubated with 50 nM of stem-loop RT primer (piR-1; 5′-GTCGTATCCAGTGCAGGGTCCGAGGTATTCGCACTGGATACGACGTTCGA-3′, piR-2; 5′-GTCGTATCCAGTGCAGGGTCCGAGGTATTCGCACTGGATACGACCCGCAG-3′), 1 × SuperScript III reaction mix (0.25 mM each dNTP, 1 × First-Strand Buffer, 5 mM DTT, and 200 U Superscript III reverse transcriptase), and 1 U of RNase inhibitor (Promega) for 30 min at 16 °C, 30 min at 42 °C, and then 15 min at 70 °C. The resultant cDNA was quantified by real-time PCR using Ssofast Evagreen Supermix (Biorad), and the specific forward primers (piR-1; 5′-CCGCTCAAAAACTAACGGATTG-3′, piR-2; 5′-CGCCAAAAGCATGAGAATTTGC-3′) and a common reverse primer (5′-GTGCAGGGTCCGAGGT-3′). The expression of sno-133 and 5 S rRNA was quantified for use as a control using the following primers: stem-loop RT primer (sno-133; 5′-GTCGTATCCAGTGCAGGGTCCGAGGTATTCGCACTGGATACGACAAACTC-3′, 5 S rRNA; 5′-GTCGTATCCAGTGCAGGGTCCGAGGTATTCGCACTGGATACGACAAGCCA-3′), forward primer (sno-133; 5′-GCCCAATTTAATGTGGAAATCTC-3′, 5 S rRNA; 5′-TAATGGTGACCGCCTGGGAACACC-3′), and the common reverse primer. The reaction mixtures were incubated at 98 °C for 30 s, followed by 40 cycles of 98 °C for 5 s and 60 °C for 5 s using the CFX96 Real-Time Detection System (Biorad). For quantification of piR-a–p (Supplementary Table S2), each of the piRNA-specific forward and stem-loop reverse primers (Supplementary Table S3) and One-Step SYBR PrimeScriptª RT-PCR Kit II (Clontech) were used. Reaction mixtures were incubated at 16 °C for 15 min, 42 °C for 15 min, and then 95 °C for 10 s, followed by 40 cycles of 95 °C for 5 s and 60 °C for 34 s using StepOne Plus Real-Time PCR machine (Applied Biosystems).

### Northern blot

Northern blot for piR-1 and piR-2 was performed as described previously^36^. BmN4 total RNA was resolved by 15% PAGE containing 7 M urea, transferred to Hybond N + membranes (GE Healthcare), and hybridized to 5′-end labeled antisense probes detecting piR-1 (5′-GTTCGAAACCAATCCGTTAGTTTTTGA-3′), piR-2 (5′-GCCGCAGACAGCAAATTCTCATGCTTTT-3′), and *Bombyx* 5 S rRNA (5′-GCTTGACTTCGGTGATCGGACGAGAAC-3′). Typhoon 9400 and ImageQuant ver. 5.2 (GE Healthcare) were used for storage phosphor autoradiography visualization, and ImageJ was used for band quantifications.

### Next-generation sequencing of RNAs

The total RNA was extracted from BmN4 cells after 30 h culture with the starting cell densities of 0.6 × 10^3^ (low), 3.0 × 10^3^ (medium), or 6.0 × 10^3^ (high) cells/cm^2^. RNAs with lengths under 150 nt were gel-purified and subjected to cDNA amplifications and next-generation sequencings using 5500xl SOLiD System (Life Technologies) at the Cancer Genomics Laboratory of the Sidney Kimmel Cancer Center of Thomas Jefferson University.

### Bioinformatics analyses

RNA seq data from BmN4 cells with low-, medium-, and high-densities contained 117,552,644, 144,877,411, and 152,257,139 raw reads, respectively, and can be found publicly at NCBI’s Sequence Read Archive (accession no. SRP104077). Before mapping, we used the cutadapt tool (http://dx.doi.org/10.14806/ej.17.1.200; http://journal.embnet.org/index.php/embnetjournal/article/view/200) to perform quality check and adapter trimming. The processed sequences were non-uniquely mapped to the *B*. *mori* genome extracted from SilkDB v2.0 (http://www.silkdb.org/silkdb/) by SHRiMP2^55^, allowing a 4% mismatch rate. No insertions or deletions were permitted and reads that mapped to >10,000 places were removed from subsequent analyses. For nucleotide composition analysis of piRNAs, 24–30-nt reads were non-uniquely mapped to 1,811 *B*. *mori* transposons^56^, and the mapped reads were applied to FastQC. Ping-pong analysis was performed by calculating distances between 5′-ends of piRNAs across opposite genomic strand as described previously^36^. The reads uniquely mapped to 1,811 *B*. *mori* transposons were used for the ping-pong analysis.

## Electronic supplementary material

SUPPLEMENTARY INFORMATION
