# Supplementary material for: Increasing cell density globally enhances the biogenesis of Piwi-interacting RNAs in Bombyx mori germ cells
Source: Sci Rep. 2017 Jun 23;7:4110. doi: 10.1038/s41598-017-04429-7 (PMC5482885; doi:10.1038/s41598-017-04429-7)
Supplement: Supplementary file 1 — SUPPLEMENTARY INFORMATION [file 41598_2017_4429_MOESM1_ESM.pdf]

SUPPLEMENTARY INFORMATION

Title: Increasing cell density globally enhances the biogenesis of Piwi-interacting RNAs in *Bombyx mori* germ cells

Author: Shozo Honda, Phillipe Loher, Keisuke Morichika, Megumi Shigematsu, Takuya Kawamura, Yoriko Kirino, Isidore Rigoutsos, and Yohei Kirino*

*Correspondence:
Yohei Kirino, Ph.D.
Computational Medicine Center
Department of Biochemistry and Molecular Biology
Sidney Kimmel Medical College
Thomas Jefferson University
1020 Locust Street, JAH Suite #M77
Philadelphia, PA 19107, USA
Tel: 215-503-8648
Email: Yohei.Kirino@jefferson.edu

SUPPLEMENTARY FIGURE LEGENDS

Figure S1. BmVasa and BmAgo3 co-localize to perinuclear granules in BmN4 cells

(A) Anti-BmVasa antibody (BmVasa571) recognized BmVasa (66 kDa) on Western blot using BmN4 total lysate. The arrow indicates the recognized protein.

(B) Immunofluorescence staining of BmN4 cells using anti-BmVasa (green) and DAPI (DNA, blue) showed localization of BmVasa in perinuclear granules.

(C) BmN4 cells stably expressing FLAG-tagged BmAgo3 [1] were subjected to immunofluorescence staining using anti-BmVasa (green), anti-FLAG (BmAgo3, red), and DAPI (DNA, blue). A merged image of the three channels is shown on the right, showing co-localization of BmVasa and BmAgo3 in perinuclear granules.

Figure S2. Quantitative ability of Western blots

(A) Varying amounts (2.5, 5, 10 and 20 μg) of total protein lysates from BmN4 high-density cells were probed on Western blots with indicated antibodies.

(B) The band intensities of Western blots were quantified using Image Lab 5.2.1 (BioRad). Proportional correlation of BmN4 lysate input to the band intensities suggested the quantitative ability of Western blots. Each data set represents the average of two independent experiments.

Figure S3. No change in total protein staining patterns among BmN4 cells with different densities

SDS-PAGE was applied to 10 μg of total protein lysates of BmN4 cells with the indicated starting densities and stained with Coomassie Blue.

Figure S4. No change in mitochondrial staining patterns among BmN4 cells with different densities

BmN4 cells with the indicated starting densities were incubated with 100 nM of MitoTracker Red CMXRos (Life Technologies) for 30 min at 27°C. After DNA counterstaining with ProLong Gold Antifade Reagent with DAPI (Life Technologies), images were acquired using a Nikon C1 confocal laser microscope. Scale bar, 10 μm. The mitochondrial staining patterns were not altered by the cell density, suggesting that cell density does not influence mitochondrial shape and morphology.

Figure S5. The rate of BmN4 cell proliferation increases as cell density increases, but cell cycle arrest does not influence the expression levels of Piwi protein

(A) BmN4 cells were plated at the represented cell density. After 24 h incubation, cell numbers were counted and cell proliferation rates were evaluated. Each data set represents the average of three independent experiments with bars showing the SD. Cell proliferation rate increased as cell density increased.

(B) To examine the influence of cell proliferation rate on Piwi protein levels, the cell cycle of BmN4 cells was arrested by double thymidine block as described previously [2]. BmN4 cells were treated with 2.5 mM thymidine for 16 h, released by washing with PBS and incubated for 9 h, and then again treated with 2.5 mM thymidine for 16 h. To confirm the cell cycle arrest, propidium iodide (PI) flow cytometric assay was performed. The cells were fixed by 70% ethanol for 2 h at -20°C, followed by treatment with 2 mg/mL of RNase A for 20 min at 37°C. Subsequently, the cells were stained with 20 μg/mL of PI for 30 min on ice, washed with PBS, and then subjected to the analyses using LSR II Flow Cytometer (BD Biosciences) at the Flow

Cytometry Facility of the Sidney Kimmel Cancer Center of Thomas Jefferson University. The data analysis of 10,000 cells using FACSDiva software (BD Biosciences) showed successful cell cycle arrest induced by double thymidine block.

(C) The cells treated with or without thymidine were subjected to Western blot with the antibodies for Piwi proteins and β-actin (control). The expression levels of Piwi proteins were not altered by the cell cycle arrest, suggesting that cell proliferation does not influence the levels of Piwi proteins.

Figure S6. Quantification of mature piRNAs by qRT-PCR using a stem-loop primer

(A) Schematic representation of the qRT-PCR using a stem-loop primer for quantification of mature piRNAs, based on the stem-loop qRT-PCR for miRNA quantification [3]. A stem-loop RT primer was specifically hybridized to the 3′-end of the target piRNA, followed by reverse transcription. The resultant cDNA was subsequently amplified and quantified.

(B) To examine the quantification ability, the stem-loop qRT-PCR for piR-1 or piR-2 quantification was applied for different amounts of BmN4 total RNA input. The quantifications showed clear linearity between the log of total RNA input and the Ct value, indicating that the stem-loop qRT-PCR is capable of quantifying mature piRNAs in total RNA.

Figure S7. Cell density-dependent change in the levels of miRNAs

The expression levels of the indicated 6 miRNAs in BmN4 cells with the 0.6 (low: L), 3.0 (medium: M), or 6.0×10^3 (high: H) cells/cm^2 starting densities were quantified by qRT-PCR. DNase-treated total RNA was subjected to reverse transcription with the following stem-loop RT primers: bmo-bantam-3p; 5′-

GTCGTATCCAGTGCAGGGTCCGAGGTATTCGCACTGGATACGACAATTAG-3′, bmo-miR-100; 5′-GTCGTATCCAGTGCAGGGTCCGAGGTATTCGCACTGGATACGACCACAAG-3′, bmo-miR-306a-5p; 5′-GTCGTATCCAGTGCAGGGTCCGAGGTATTCGCACTGGATACGACTCAGAG-3′, bmo-miR-279b-3p; 5′-GTCGTATCCAGTGCAGGGTCCGAGGTATTCGCACTGGATACGACTCAATG-3′, bmo-miR-278-3p; 5′-GTCGTATCCAGTGCAGGGTCCGAGGTATTCGCACTGGATACGACAAACGG-3′, and bmo-miR-14-3p; 5′-GTCGTATCCAGTGCAGGGTCCGAGGTATTCGCACTGGATACGACTAGGAG-3′. The resultant cDNA was quantified by real-time PCR using the specific forward primers (bmo-bantam-3p; 5′-GGCGTGAGATCATTGTGAA-3′, bmo-miR-100; 5′-GACGAACCCGTAGATCCG-3′, bmo-miR-306a-5p; 5′GGCGTCAGGTACTAGGT-3′, bmo-miR-279b-3p; 5′-CGGCGTGACTAGATCTACA-3′, bmo-miR-278-3p; 5′-GACGTCGGTGGGATCTTC-3′, and bmo-miR-14-3p; 5′-CGGCGTCAGTCTTTTTCT-3′) and a common reverse primer (5′-GTGCAGGGTCCGAGGT-3′). The expression of 5S rRNA was used as an internal control. Expression levels in the low-density cells were set as 1. Each data set represents the average of three independent experiments with bars showing the SD.

Figure S8. No big differences in alignment visualization patterns of transposon-mapped piRNAs among BmN4 cells with different densities

The piRNA-enriched 24–30-nt reads from BmN4 cells with the 0.6 (low), 3.0 (med), or 6.0 × 10^3 (high) cells/cm^2 starting densities were non-uniquely mapped to 1,811 *B. mori* transposons [4] using Bowtie program [5] and visualized using Tablet (https://ics.hutton.ac.uk/tablet/). The alignment visualization patterns of piRNAs for top eight most abundantly-mapped 5–8 kb transposons (high-density data) were shown. The sequences mapped to sense and anti-sense strand are represented in green and blue, respectively.

SUPPLEMENTARY TABLES

Table S1. Primer sequences for qRT-PCR to quantify mRNAs, piRNA precursor, and transposons

Target	Primer sequence (5′-3′)
BmRp49-F	GGATCGCTATGACAAACTTAAGAGG
BmRp49-R	TATGACGGGTCTTCTTGTTGGA
Siwi-F	GTGTCGGTGATGGTCAAATCC
Siwi-R	TGCCAACTTTATTTCGACTCCTG
BmAgo3-F	GTTGTTCACTCAGAGGGATAAGCA
BmAgo3-R	CAGCGTCAGAAACCATAATACCC
BmVasa-F	TTAGATATGGGGTTCATGCCAAG
BmVasa-R	ATTTAGGAAACGACCAGCCAAA
BmPapi-F	ACAGATTGAAGTCGCCAAGAAAC
BmPapi-R	CGCTTCGACTGCTCTATCTCC
RpL3-F	GCGCTCTGTTGACTGTCTGG
RpL3-R	TGCCATTTCTTACTGGCTTTAGTG
ActinA3-F	GTATGTGCAAGGCCGGTTTCG
ActinA3-R	TCCTTCTGTCCCATGCCGAC
Torimochi-F	AGCTCATCGCCGTATCTTGAAGA
Torimochi-R	TGGATATAGTATGTGGATACAG
Yamato-F	AAGAAGCGAACCCGGTACTTG
Yamato-R	GGTGAGCAGGAAGTCGTTTG
Kimono-F	GCGGACCGAGTAAATATTAGCAG
Kimono-R	TGGCAGGCATGAGTTACAAAG

Table S2. Sequences of mature piR-a–p

piRNA	Sequence (5′-3′)
piR-a	CGACAGTTCTTCTTTAAATTTTCCCACT
piR-b	TCCACACTTGAACTTTGTACTCTCGACC
piR-c	TCCCTGCAGAAGGAACGGCCTCCTCATC
piR-d	AATCCAACAGAGCTCGAGCAAACACAGT
piR-e	GCGAAAATTGGATCTTGCGTTTCCTGT
piR-f	TCGCTATAGAATTCAATTCTGTACCCGC
piR-g	TCCCATATGGCAATAAATGCATCGTAC
piR-h	AATCGTCCTGTGAAGCCTAGGTGTCAT
piR-i	TCCTCTAGTTTTATGAATATTATCCGTC
piR-j	TGACTCAACTTGTTACTAGAACCCTGTC
piR-k	TACCATACGCAACTTCAATGTTGGGACC
piR-l	ATGACATCGAAAGCTATTTTCTTCCAG
piR-m	TGCTATTCTCTATCAATTCTATCAACGC
piR-n	TCAACACAATCTTCAGCCTGATAGGTGC
piR-o	AGACAACCGATCGTACACTACCTCCAAC
piR-p	TCATACTTGAAGTCGGCACAATTCCACTC

Table S3. Primer sequences for qRT-PCR to quantify mature piRNAs

Target	Primer sequence (5′-3′)
piR-a	F: GCCGACAGTTCTTCTTTAAATTTTC<br>R: GTCGTATCCAGTGCAGGGTCCGAGGTATTCGCACTGGATACGACAGTGGG
piR-b	F: GCTCCACACTTGAACTTTGTACTC<br>R: GTCGTATCCAGTGCAGGGTCCGAGGTATTCGCACTGGATACGACGGTCGA
piR-c	F: TCCCTGCAGAAGGAACG<br>R: GTCGTATCCAGTGCAGGGTCCGAGGTATTCGCACTGGATACGACGATGAG
piR-d	F: GGAATCCAACAGAGCTCGA<br>R: GTCGTATCCAGTGCAGGGTCCGAGGTATTCGCACTGGATACGACACTGTG
piR-e	F: GCGAAAATTGGATCTTGCG<br>R: GTCGTATCCAGTGCAGGGTCCGAGGTATTCGCACTGGATACGACACAGGA
piR-f	F: GCGGTAGAATTCAATTCTGTAC<br>R: GTCGTATCCAGTGCAGGGTCCGAGGTATTCGCACTGGATACGACGCGGGT
piR-g	F: CCCATATGGCAATAAATGCA<br>R: GTCGTATCCAGTGCAGGGTCCGAGGTATTCGCACTGGATACGACGTACGA
piR-h	F: AATCGTCCTGTGAAGCCTAG<br>R: GTCGTATCCAGTGCAGGGTCCGAGGTATTCGCACTGGATACGACATGACA
piR-i	F: GCCGTCCTCTAGTTTTATGAATATTATC<br>R: GTCGTATCCAGTGCAGGGTCCGAGGTATTCGCACTGGATACGACGACGGA
piR-j	F: GCGGTGACTCAACTTGTTACTAGA<br>R: GTCGTATCCAGTGCAGGGTCCGAGGTATTCGCACTGGATACGACGACAGG
piR-k	F: TACCATACGCAACTTCAATGTTG<br>R: GTCGTATCCAGTGCAGGGTCCGAGGTATTCGCACTGGATACGACGGTCCC
piR-l	F: GCATGACATCGAAAGCTATTTTC<br>R: GTCGTATCCAGTGCAGGGTCCGAGGTATTCGCACTGGATACGACCTGGAA
piR-m	F: GCATGCTATTCTCTATCAATTCTATCA<br>R: GTCGTATCCAGTGCAGGGTCCGAGGTATTCGCACTGGATACGACGCGTTG
piR-n	F: TCAACACAATCTTCAGCCTGATA<br>R: GTCGTATCCAGTGCAGGGTCCGAGGTATTCGCACTGGATACGACGCACCT
piR-o	F: AGACAACCGATCGTACACTACC<br>F: GTCGTATCCAGTGCAGGGTCCGAGGTATTCGCACTGGATACGACGTTGGA
piR-p	F: TCATACTTGAAGTCGGCACAAT<br>R: GTCGTATCCAGTGCAGGGTCCGAGGTATTCGCACTGGATACGACGAGTGG

SUPPLEMENTARY REFERENCES

1. Honda, S. et al. Mitochondrial protein BmPAPI modulates the length of mature piRNAs. *RNA* **19**, 1405-18 (2013).
2. Honda, S. et al. Sex hormone-dependent tRNA halves enhance cell proliferation in breast and prostate cancers. *Proc Natl Acad Sci U S A* **112**, E3816-25 (2015).
3. Chen, C. et al. Real-time quantification of microRNAs by stem-loop RT-PCR. *Nucleic Acids Res* **33**, e179 (2005).
4. Osanai-Futahashi, M., Suetsugu, Y., Mita, K. & Fujiwara, H. Genome-wide screening and characterization of transposable elements and their distribution analysis in the silkworm, Bombyx mori. *Insect Biochem Mol Biol* **38**, 1046-57 (2008).
5. Langmead, B., Trapnell, C., Pop, M. & Salzberg, S.L. Ultrafast and memory-efficient alignment of short DNA sequences to the human genome. *Genome Biol* **10**, R25 (2009).

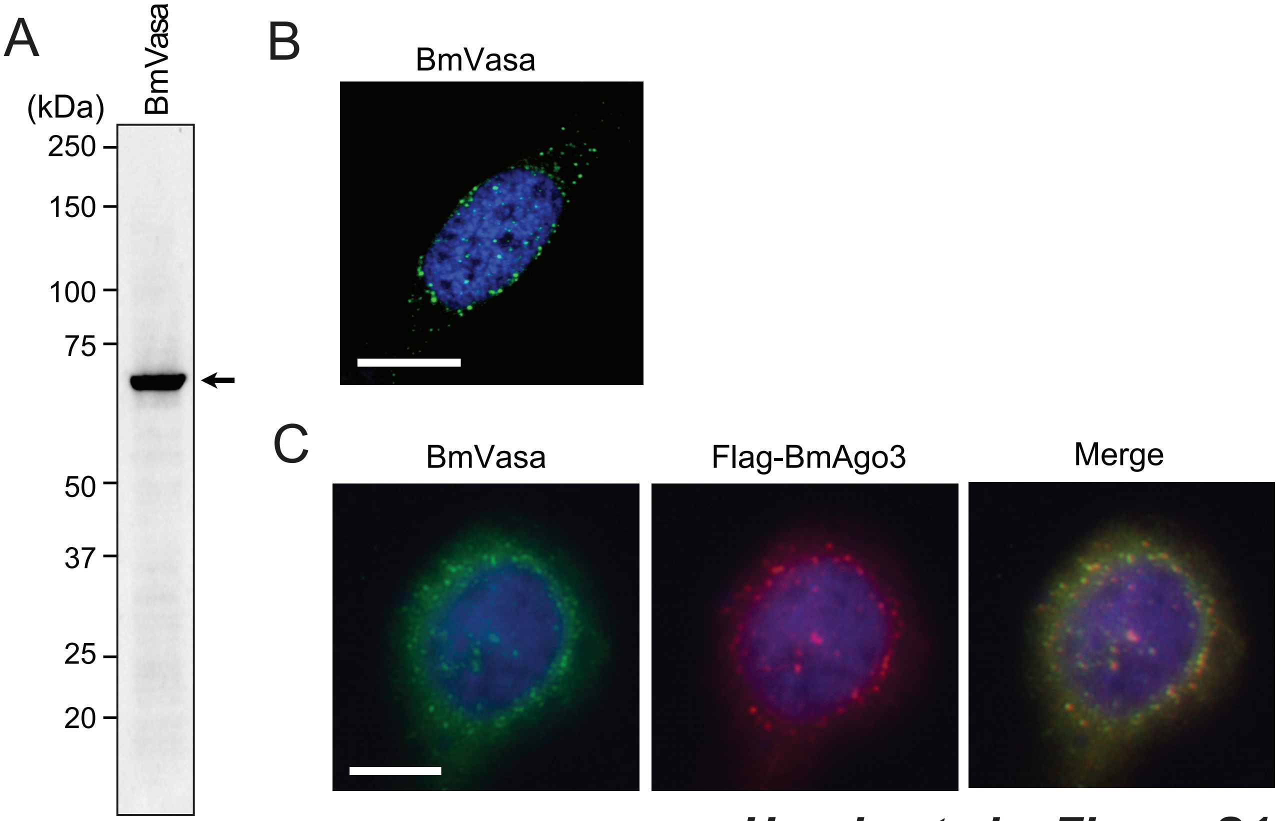

Honda et al. Figure S1

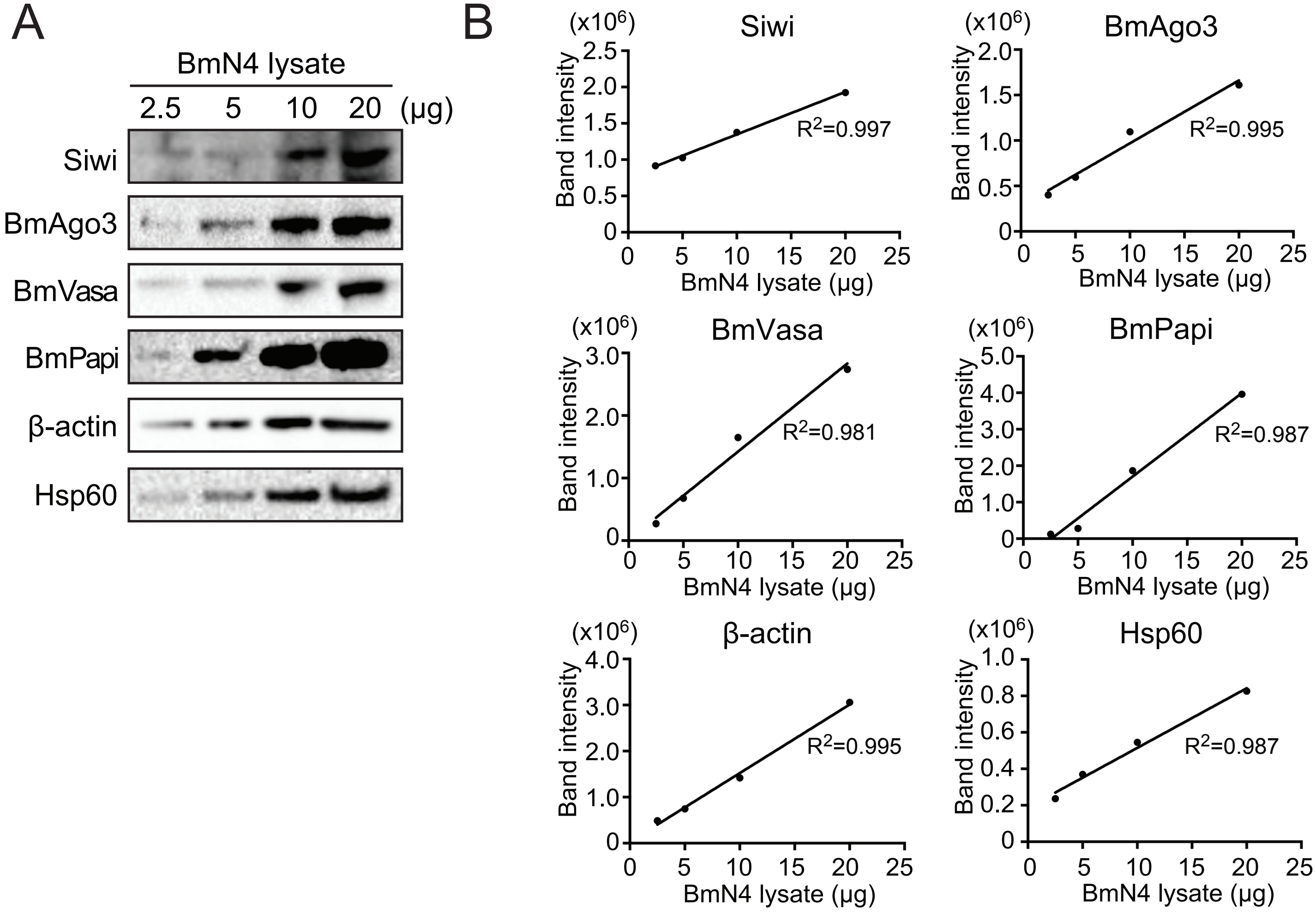

Honda et al. Figure S2

Starting cell density
(x10^{3}cells/cm^2)

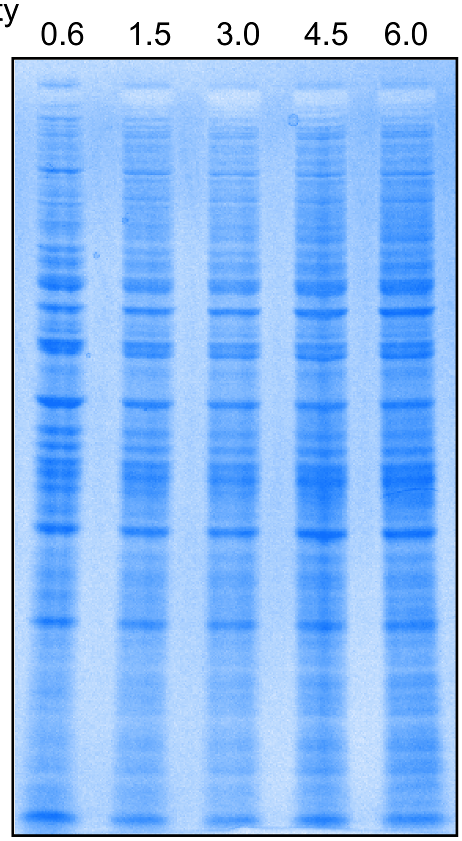

Honda et al.   Figure S3

Starting cell density
(x10^{3}cells/cm^2)

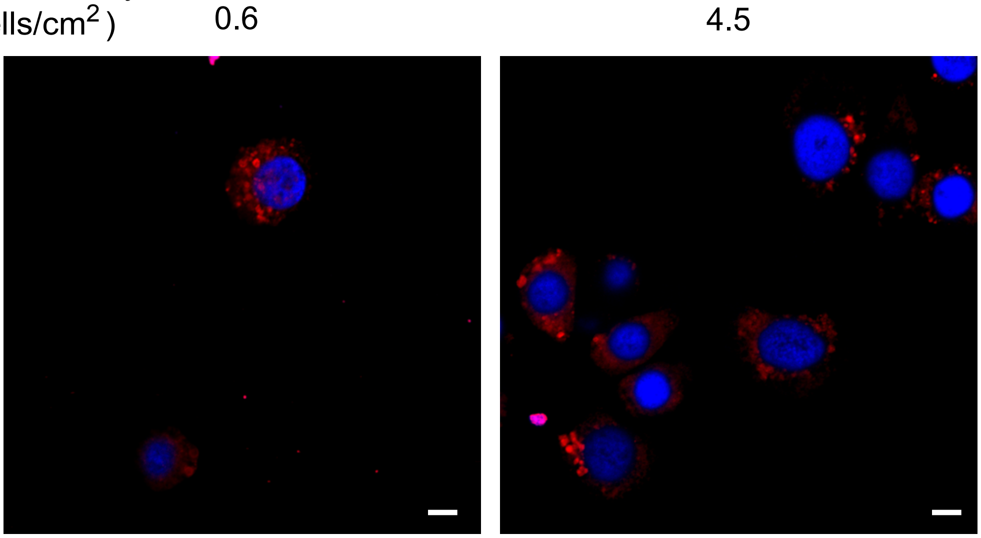

Honda et al.   Figure S4

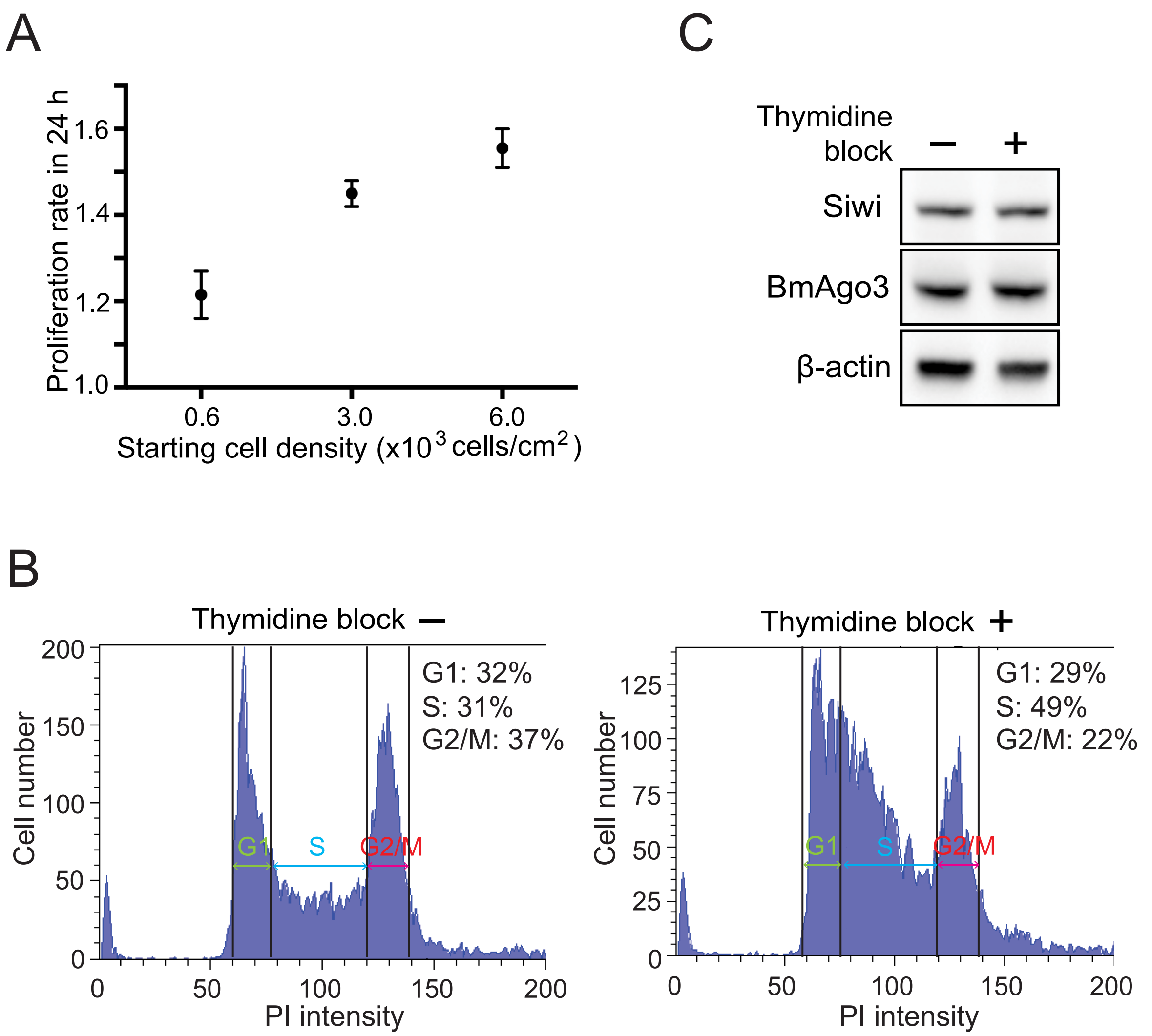

Honda et al. Figure S5

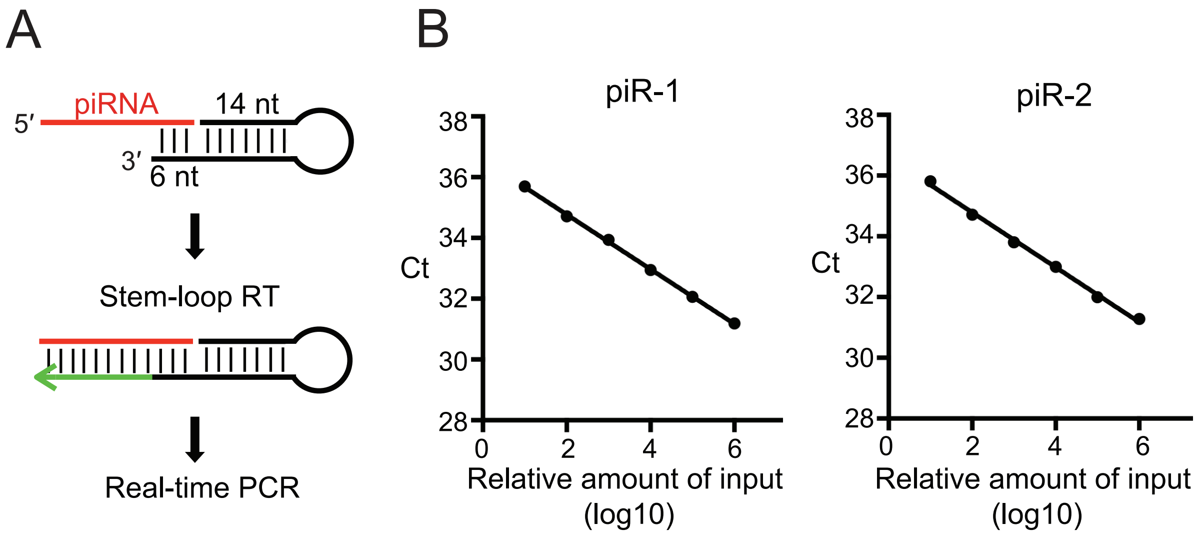

Honda et al. Figure S6

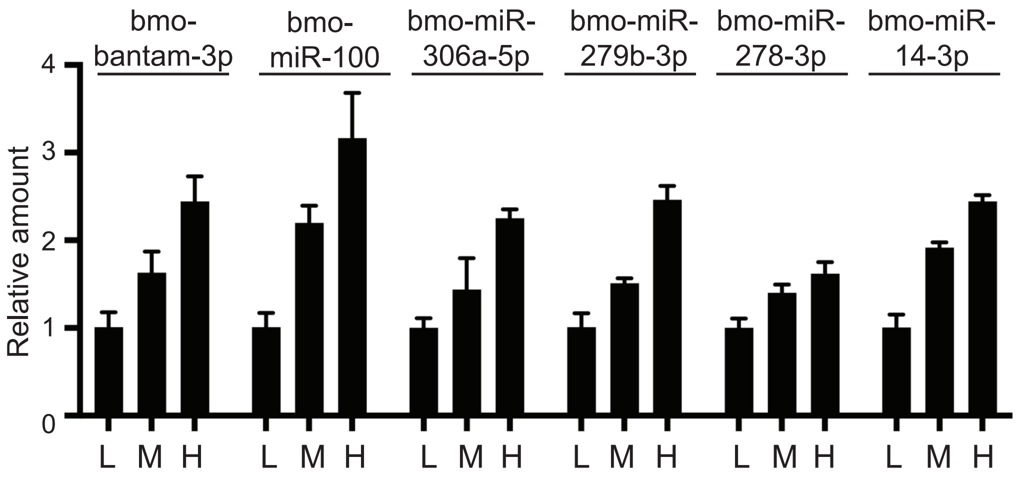

Honda et al. Figure S7

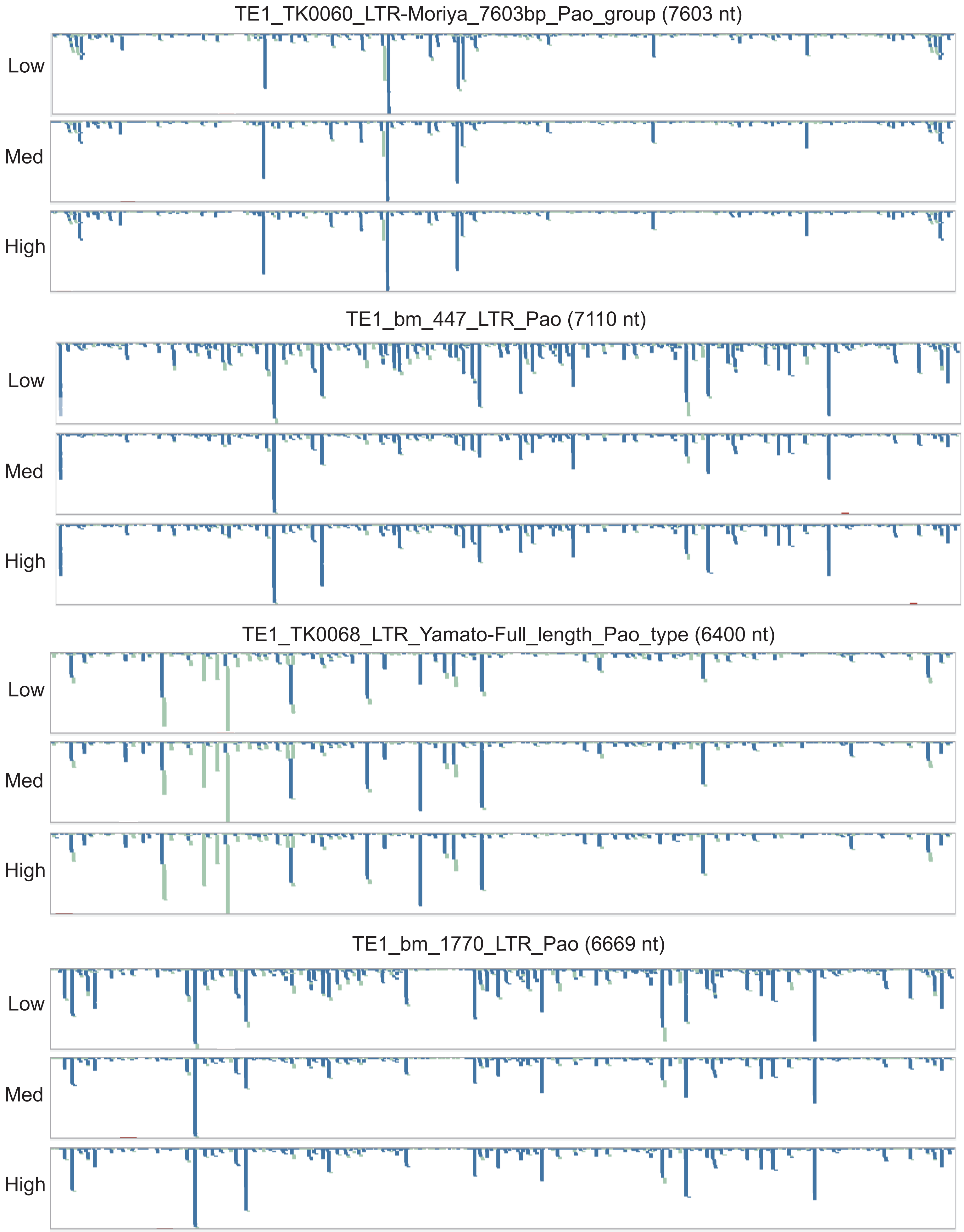

Honda et al. Figure S8-1

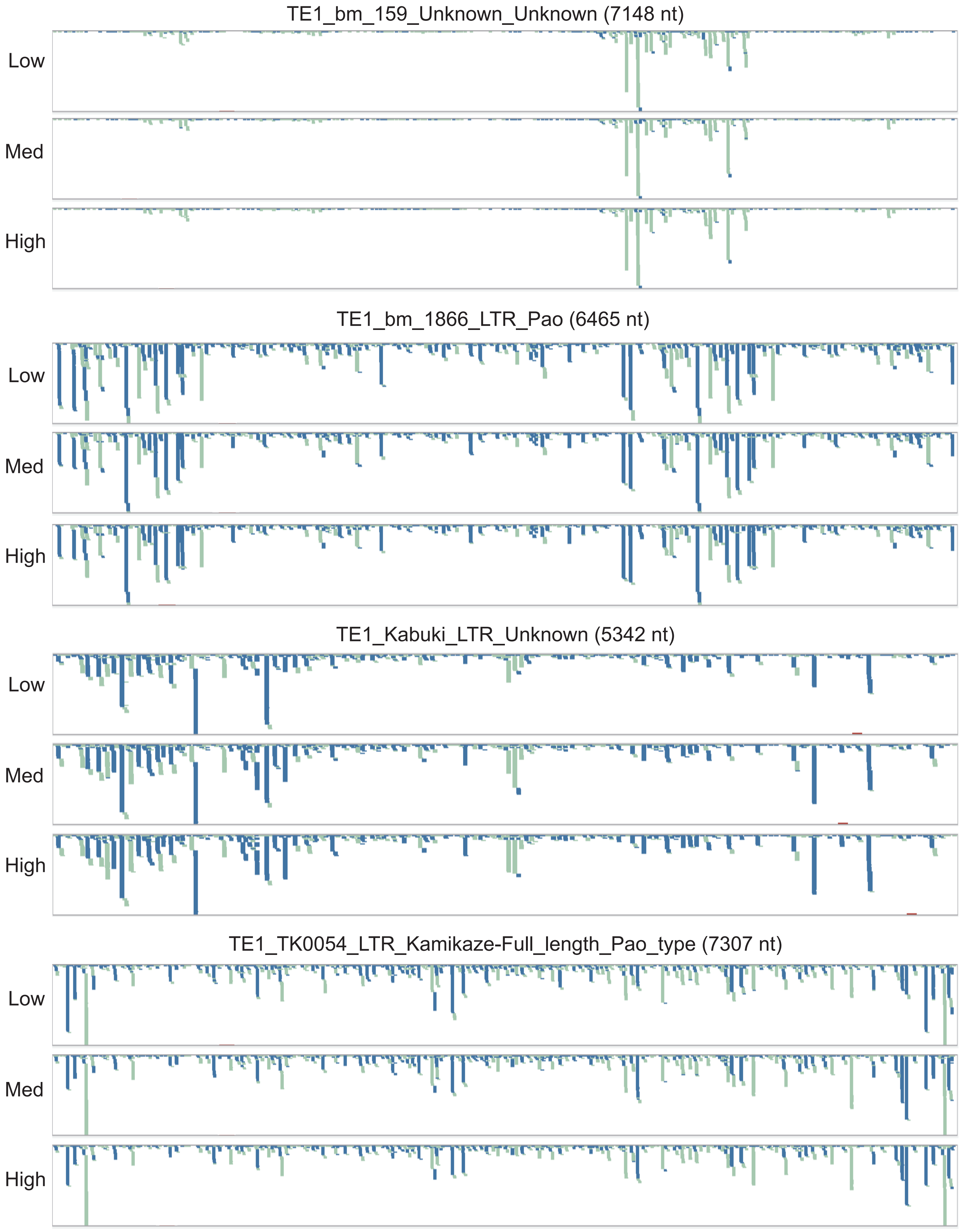

Honda et al. Figure S8-2
